# Human Fungal Infections: Diagnostic and Therapeutic Challenges

**DOI:** 10.3390/jof11010066

**Published:** 2025-01-16

**Authors:** Timoleon-Achilleas Vyzantiadis, Eleni Gavriilaki, Athanasios Tragiannidis

**Affiliations:** 1 Department of Microbiology, Medical School, Aristotle University of Thessaloniki, 54124 Thessaloniki, Greece; 2 Second Propedeutic Department of Internal Medicine, Hippokration Hospital, Aristotle University of Thessaloniki, 54642 Thessaloniki, Greece; gavriiel@auth.gr; 3 Childhood & Adolescent Haematology Oncology Unit, Second Department of Paediatrics, AHEPA Hospital, Aristotle University of Thessaloniki, 53646 Thessaloniki, Greece; atragian@auth.gr

Fungal infections, either invasive and disseminated or cutaneous and superficial, are an important and increasing cause of morbidity [1,2,3,4].

The first category consists of a major factor of severe disease and mortality, especially in immunocompromised and debilitated patients. However, the second category can also cause serious concerns in matters of human health, not forgetting that it is extremely widespread [5].

Several causes [6] like the broadly used chemotherapies and immunosuppressive treatments, haematopoietic cell transplantation and novel cellular therapies, several iatrogenic interventions, the ageing of the population, severe underlying diseases, mass migration and excessive travelling, changing of social habits, broad climate change, epidemiology alteration in terms of fungal species and antifungal sensitivities, co-infections and super-infections, emerging and new fungal pathogens are all factors that make fungal infections an important issue of public health at both the community and hospital levels.

In addition, fungi are a complex and greatly evolved kingdom of living entities that are very adept at surviving and expanding. The eukaryotic organisation and the resistance of their cells and the multitude of invading and escaping mechanisms they possess make them a serious opponent in terms of disease.

All the above are important challenges in the diagnostic procedure [7,8] but also in the treatment of the relevant infections in both the paediatric and the adult populations [9,10,11].

Moreover, since the onset of the COVID pandemic, it was realised, once more, how vulnerable we become when a second invader, like the fungi, takes advantage of the exhausting of the human organism’s resistance resources and how much more difficult diagnosis and treatment can become. The last was extensively analysed in this Special Issue by Contribution 1.

The specific Special Issue in total had the ambition to focus on these challenges and discuss several aspects of diagnostic and therapeutic approaches for the two types of fungal infection, invasive or superficial.

Together with the presentation of single (by Contribution 2, Contribution 3, and Contribution 4) or series (by Contribution 5) of interesting and challenging cases that concerned the diagnostic and therapeutic approaches for specific patients, the description of fungal infections in specific clinical entities wasalso fully described concerning nail psoriasis by Contribution 6 and acute lymphoblastic leukaemia by Contribution 7 Comparisons of different methods for the measuring of galactomannan by Contribution 8 and a study on long-term kinetics of serum galactomannan in two severely immunocompromised adolescents by Contribution 9 showed the constant effort concerning the applied methodology as well as the clinical usefulness of fungal biomarkers. Also, the major challenge of alternative or new antifungal agents was faced in the case of protection from vulvovaginal candidiasis by Contribution 10 and in the case of infection by *Candida auris* in the submission by Contribution 11. Finally, the major issue of antifungal resistance, mainly for dermatophytic infections, was reviewed in the context of the formation of relevant biofilms by Contribution 12.
